# Challenging the limits of mitral-TEER: a PASCAL case in the anatomical ‘red zone’: a case report

**DOI:** 10.1093/ehjcr/ytag630

**Published:** 2026-08-27

**Authors:** Konstantinos Papadopoulos, Laila S Khan, Apostolos Tzikas, Alina-Cristina Badescu, Mani A Vannan

**Affiliations:** Echocardiography Laboratory, European Interbalkan Medical Center, Asklipiou 10 str, Pylaia, Thessaloniki 57001, Greece; Department of Cardiology, The Canberra Hospital, Canberra Health Services, Canberra, Yamba Drive, Garran, ACT 2605, Australia; Second Department of Cardiology, Hippokration University Hospital, Aristotle University of Thessaloniki, Konstantinoupoleos 49, Thessaloniki 54642, Greece; Cardiology Department, European Interbalkan Medical Center, Asklipiou 10 str, Pylaia, Thessaloniki 57001, Greece; Faculty of Medicine, Ovidius University of Constanta, 124 Mamaia Boulevard, Constanta 900527, Romania; Structural and Valvular Center of Excellence, Marcus Heart Valve Center, Piedmont Heart Institute, 95 Collier Rd NW Ste 5015, Atlanta, GA 30309, USA

**Keywords:** Mitral regurgitation, PASCAL, TEER, Edge-to-edge repair, Heart failure, Echocardiography, Case report

## Abstract

**Background:**

Transcatheter edge-to-edge repair (TEER) has emerged as an effective therapeutic option for patients with severe mitral regurgitation (MR) deemed high risk for surgery. Two devices are currently available in Europe, MitraClip (Abbott Vascular, Santa Clara, CA, USA) and PASCAL (Edwards Lifesciences, Irvine, CA, USA). The PASCAL system provides unique features, such as a central spacer and nitinol flexibility, that may expand eligibility in anatomically and clinically challenging cases.

**Case summary:**

We present the case of a 78-year-old female with ischaemic cardiomyopathy and advanced heart failure (HF) [ejection fraction (EF) of 25%), severe secondary MR (SMR), chronic kidney disease, and recurrent pulmonary oedemas with hospitalizations with an STS score of 26.8%]. Thus, transcatheter TEER was deemed the best option. The baseline mitral valve area (MVA) and mean gradient were 2.7 cm^2^ and 2.4 mmHg, respectively. Therefore, TEER with the PASCAL P10 device was selected as the most optimal device. A single device was implanted at the A_2_-P_2_ scallops, resulting in significant MR reduction and an acceptable final mean gradient of 4.5 mmHg. The patient was discharged clinically improved and without any hospitalizations for heart failure in 1-year follow up period.

**Discussion:**

This case illustrates the feasibility of TEER with PASCAL in a frail, high-risk patient with severe SMR and borderline anatomy. The spacer technology and leaflet-friendly design allowed safe and effective MR reduction while maintaining acceptable gradients. This case highlights the importance of individualized heart team decision-making, the potential advantages of the PASCAL device in anatomically challenging scenarios and increased operator experience.

Learning points

- Careful echocardiographic assessment of mitral valve anatomy and annulus dimensions can identify borderline patients who may benefit from TEER.
- Individualized device selection can achieve effective MR reduction while maintaining acceptable *trans*-mitral gradients in complex SMR anatomies.

## Introduction

Mitral regurgitation (MR) is a common heart-valve disease worldwide. Secondary MR (SMR) is associated with poor prognosis in patients with heart failure (HF).^1^ Surgical repair has been associated with high rates of MR recurrence while recent studies have shown that transcatheter edge-to-edge repair (TEER) provides comparable results to surgical replacement with fewer complications.^2^ Randomized trials showed TEER efficacy in SMR, resulting in updated guideline recommendations, and mitral transcatheter edge-to-edge repair (M-TEER) now has a class of recommendation I and level of evidence A (IA) indication for SMR patients that fulfil specific clinical and echocardiographic criteria and do not need revascularization.^3^ The TEER has emerged as a therapeutic alternative, initially with the MitraClip system (Abbott Vascular, Santa Clara, CA, USA), and more recently with the PASCAL device (Edwards Lifesciences, Irvine, CA, USA), which incorporates different design elements that may improve outcomes in complex anatomies.^4^

We report a frail patient with severe SMR who underwent successful TEER with PASCAL, despite borderline anatomy, highlighting the role of advanced echocardiographic imaging, multidisciplinary evaluation and tailored device selection.

## Summary figure

**Figure:**
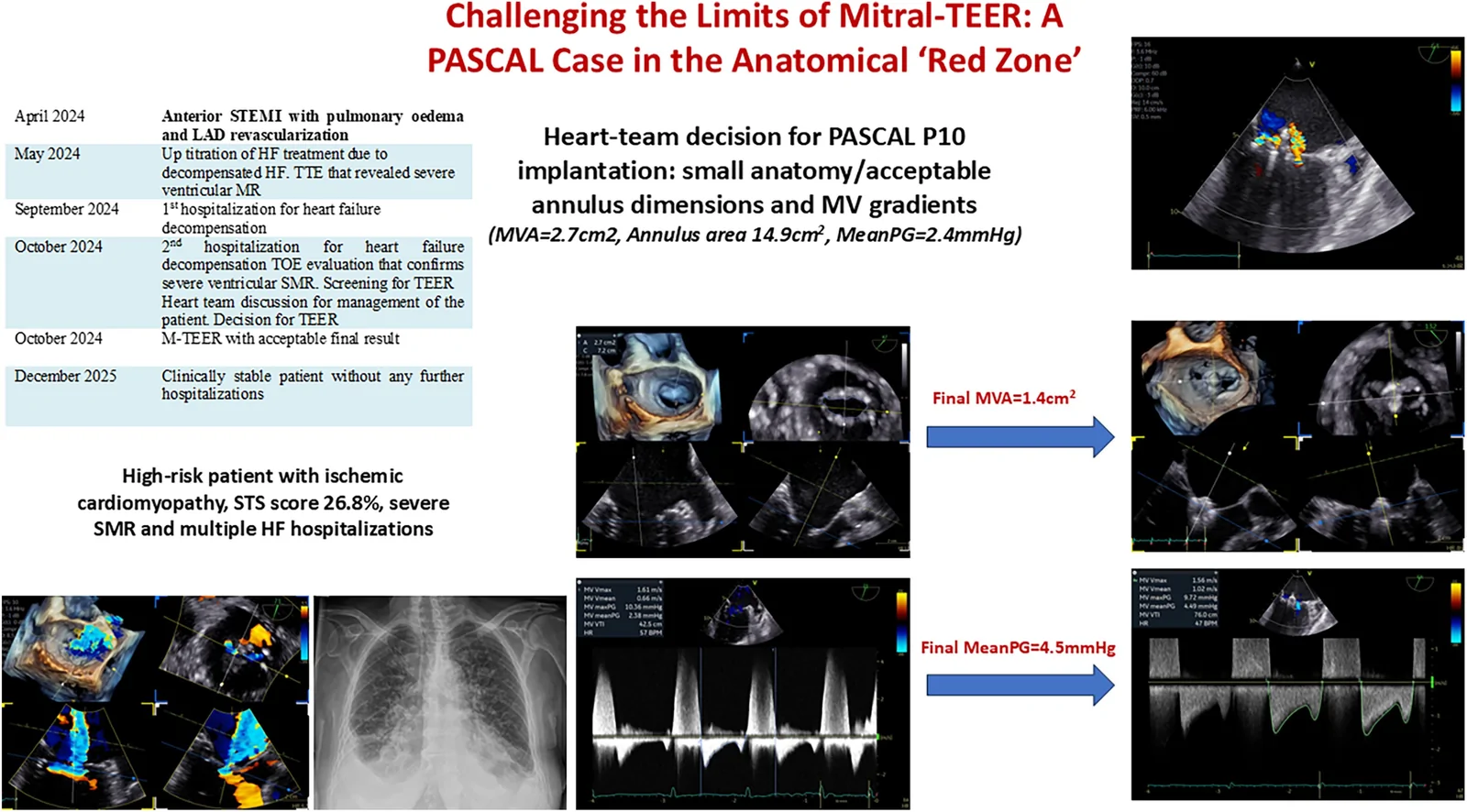


## Case presentation

A 78-year-old female with a history of anterior ST-elevation myocardial infarction (STEMI) 5 months prior (treated with delayed stenting of the left anterior descending artery (LAD)), paroxysmal atrial fibrillation, and stage IV chronic kidney disease (estimated glomerular filtration rate (eGFR) 20–30 mL/min/1.73 m^2^) was repeatedly hospitalized for decompensated HF despite guideline-directed medical therapy. Transthoracic echocardiography revealed akinesia of the anterior wall, septum and all apical segments, severely dilated left ventricle (LV) (4DLVEDVi = 153 mL/m^2^), severely reduced LV ejection fraction (25%), and concomitant severe SMR due to leaflet tethering (tenting height = 13 mm) with effective regurgitant orifice area (EROA) 40 mm^2^, regurgitant volume (RV) 53 mL, and elliptic shape of vena contracta (VC) (two-chamber width 6 mm, three-chamber width 22 mm). Laboratory testing revealed markedly elevated N-terminal pro-B-type natriuretic peptide (NT-proBNP) levels (42 908 pg/mL) likely reflecting a combination of impaired renal clearance, advanced HF and severe MR.

**Figure 1:**
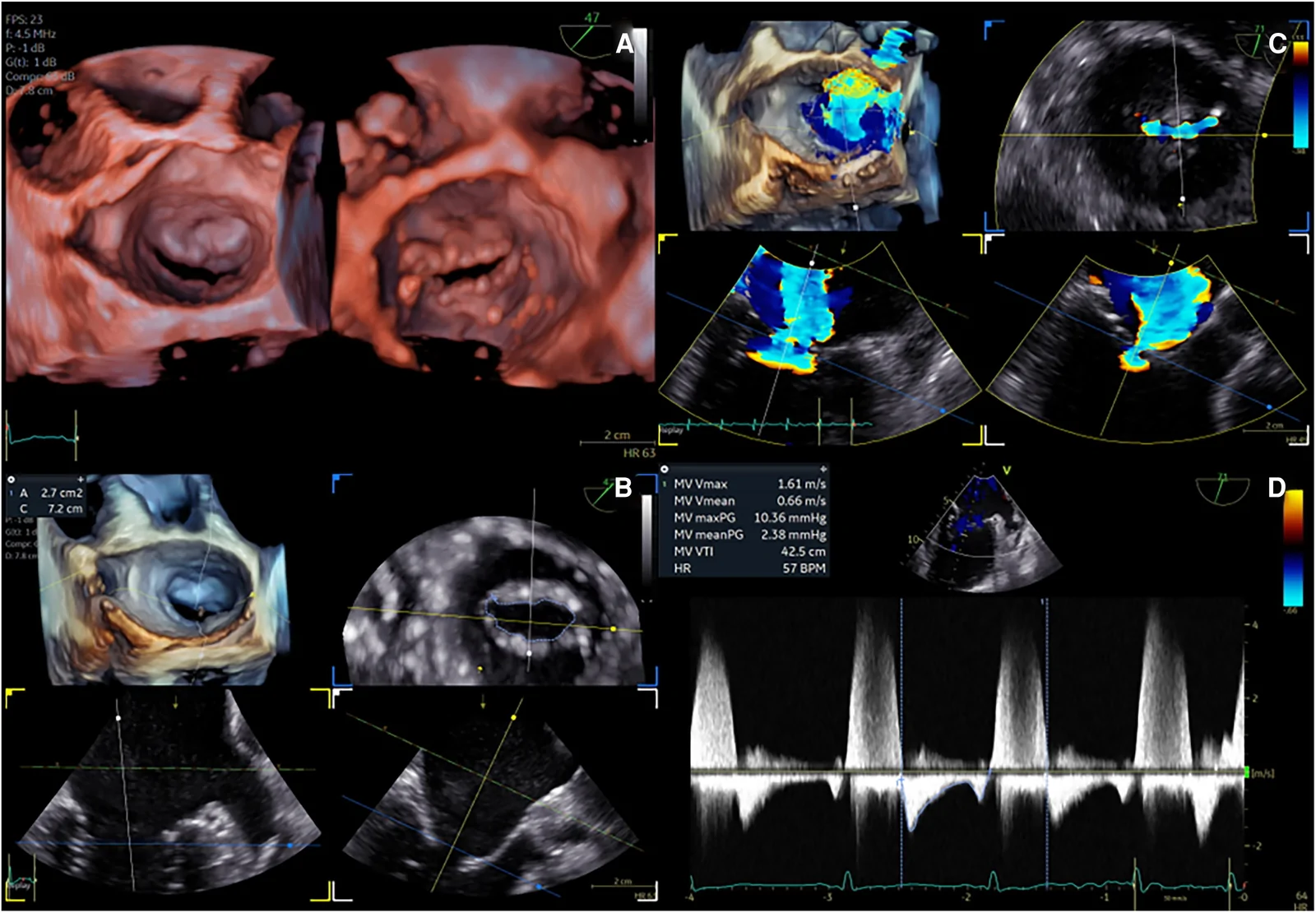
(*A*) 3D render dual crop view of mitral valve, (*B*) multi-planar reconstruction of baseline mitral valve area (2.7 cm^2^), (*C*) 3D vena contracta area, (*D*) baseline mean PG = 2.4 mmHg.

Given the high surgical risk (STS score 26.8%), the patient was considered inoperable and was referred for evaluation for anatomical suitability for TEER. Three-dimensional (3D) transoesophageal echocardiography (TOE) demonstrated severe SMR with a 3D vena contracta area (VCA) of 80 mm^2^ and MVA of 2.7 cm^2^. The patient was considered challenging for TEER due to the small anatomy, but with low *trans*-mitral gradient (mean pressure gradient (PG) = 2.4 mmHg), and acceptable mitral annulus dimensions (anterior–posterior = 3.6 cm, medial–lateral = 4.4 cm, and annulus area = 14.9 cm^2^) favouring the feasibility of the intervention (*Figure 1*). All other echocardiographic parameters were suitable for TEER (posterior leaflet length 17 mm, no cleft or multiple indentations, no calcification of leaflet tips or annulus). After discussion with the heart team and the patient regarding procedural risks and benefits, the consensus was to proceed with TEER, to reduce MR and improve quality of life.

**Figure 2:**
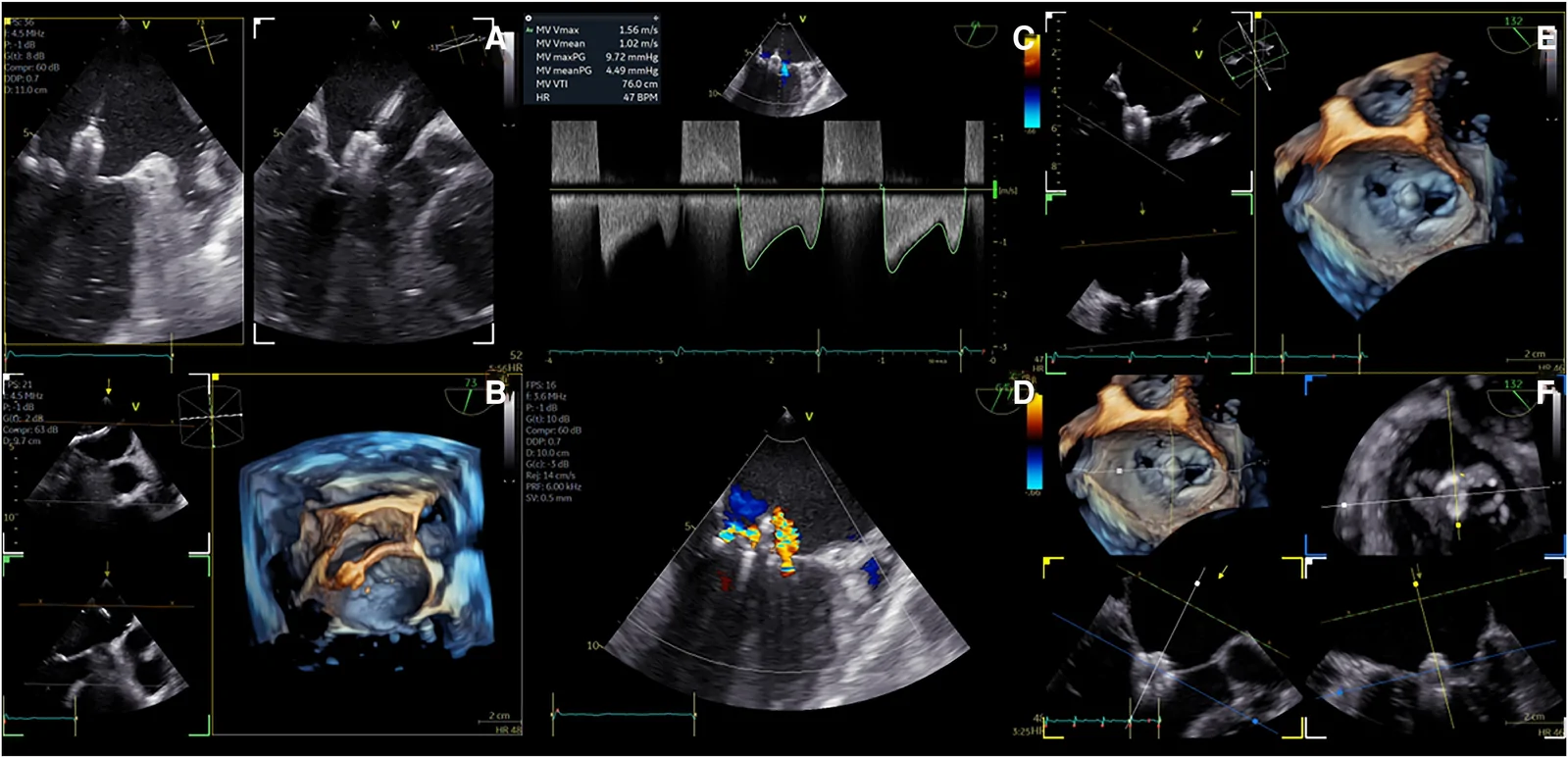
(*A*) Biplane guidance of PASCAL device, (*B*) 3D render view of PASCAL device into the left atrium, (*C*) final mean PG = 4.4 mmHg, (*D*) final mitral regurgitation, (*E*) final 3D en face view of mitral valve after TEER, *F*) Multiplanar reconstruction of final MV area.

A PASCAL P10 device was selected due to its spacer, leaflet-friendly design, and the limited closing force due to nitinol. Based on baseline TOE, the operators opted for single-device implantation in the central part of the valve with a 12-to-6‘o clock orientation, in order to minimize mitral stenosis risk. One device was successfully deployed at the A_2_-P_2_ scallops with secure leaflet grasping. Post-procedural echocardiography demonstrated significant MR reduction (EROA 10 mm^2^, RV 18 mL, VCA 20 mm^2^) with a mean gradient of 4.5 mmHg (*Figure 2*). The patient was discharged 2 days later in stable condition.

At 1-month follow-up, echocardiography showed a stable PASCAL device, with residual grade 2+ MR and a mean PG of 5 mmHg. The patient reported significant clinical improvement, and NT-proBNP levels decreased from 42.908 to <20.000 pg/mL, indicating a significant reduction consistent with clinical and haemodynamic improvement despite persistently elevated absolute values. The TEER procedure also improved cardiac output and renal perfusion leading to reduction of creatinine levels (from 4.2 to 3.2 mg/dL, BUN from 209 to 134 mg/dL) and improvement in eGFR (from 6 to 14 mL/min/1.73 m^2^).

The patient remains clinically improved in NYHA class II status, without any HF hospitalizations 1-year post-procedure and with an acceptable result of moderate MR (EROA 10 mm^2^, RV 20 mL) with MV mean PG = 5 mmHg. Renal function has remained stable without the need for haemodialysis and with creatinine level of 2.9 mg/dL and eGFR 16 mL/min/1.73 m^2^.

## Discussion

This case highlights important clinical and echocardiographic considerations for TEER patient selection. According to the EVEREST criteria,^5^ patients with a small MVA (<4.0 cm^2^, and particularly <3.0 cm^2^) are considered at higher risk of significant mitral stenosis following TEER. In our patient, 3D-TOE demonstrated a MVA of 2.7 cm^2^, representing borderline or unfavourable anatomy for TEER. Despite this limitation, annulus dimensions and the low mean *trans*-mitral gradient indicated potential feasibility of the intervention.

In this frail patient, the primary therapeutic goal was reduction of HF hospitalizations rather than restoration of completely normal valve haemodynamics. It was acknowledged that a moderate post-procedural gradient (even up to 6–7 mmHg) would be acceptable, if MR reduction was substantial and symptoms improved. Given her age, limited mobility, and poor baseline functional status, the risk of mild post-procedural stenosis was considered clinically acceptable compared with recurrent hospitalizations caused by severe MR.

The literature supports caution in “mixed” MV disease, where SMR coexists with small valve orifices.^6^ In such scenarios, TEER may create iatrogenic mitral stenosis, especially with multiple devices. However, previous studies suggest that in carefully selected patients, significant MR reduction with modestly elevated gradients may still provide clinical benefit in quality of life and HF outcomes.^7,8^ In borderline anatomies, mitral annular dimensions and calcification (MAC) may predict post-TEER stenosis. A prior computed tomography-based study proposed cut-off values (mitral annulus area <900 mm^2^, anterior–posterior diameter <2.9 cm, medial–lateral diameter <3.5 cm, MVA<2.4 cm^2^) that, when combined with a small mitral orifice, may predict high post-procedural gradients.^9^ The presence of MAC was also found to be a predictor of high post-procedural gradients after TEER.^10^ In our case, annular dimensions were within the normal range, and no calcification was identified supporting feasibility.

Large randomized trials provide important context. The COAPT trial^11^ demonstrated reduced hospitalizations and improved survival in patients with HF and severe SMR The RESHAPE-HF2 study^12^ reported similar results in patients with moderate SMR (median EROA = 25 mm^2^) with significant reduction in the composite endpoint of HF hospitalizations and mortality. More recently, the CLASP IID/IIF trial^13^ provided evidence for PASCAL, demonstrating non-inferiority compared with MitraClip regarding safety and effectiveness, while suggesting lower *trans*-mitral gradients, due to the device’s nitinol design. Importantly, 13.3% of CLASP IID/IIF patients had MVA<4.0 cm^2^ and most still achieved MR ≤ 2+ at 2 years, while 84% remained in NYHA class I status with stable gradients. A recent meta-analysis comparing MitraClip and PASCAL also demonstrated lower discharge *trans*-mitral gradient with PASCAL.^14^

In this context, the choice of the PASCAL device was particularly advantageous. Compared to MitraClip, the two available PASCAL devices (ACE and P10) incorporate a central spacer which reduces the regurgitant orifice without requiring excessive leaflet approximation, thus preserving the remaining MVA and minimizing the increase in gradients. The flexible nitinol design and broader paddles allow a lower closure force and a more physiological leaflet stress distribution, which is beneficial in borderline anatomies.^13^ Furthermore, TEER with PASCAL also appears to induce less annular remodelling than MitraClip,^15^ an additional advantage in small anatomies.

Therefore, despite borderline anatomy, careful heart team evaluation and the tailored selection of the PASCAL device allowed safe and effective MR reduction with an acceptable final gradient of 4.5 mmHg.

## Conclusion

This case highlights an individualized approach to TEER in complex SMR, emphasizing comprehensive MV and annular assessment beyond standard eligibility criteria. Careful echocardiographic evaluation can refine patient selection and guide appropriate device choice, even in borderline anatomies. Heart team discussion remains essential for integrating imaging findings into clinical decision-making and optimizing outcomes.

## Patient’s perspective

Before the procedure, I was repeatedly admitted to the hospital because of severe breathlessness and fluid in my lungs. I could no longer carry out my normal daily activities and was worried because I had been told that heart operation would be of very high risk. After the procedure, my breathing improved considerably, and I have not required further hospitalizations. I am now able to perform my daily activities with much greater ease. I am grateful to this team for offering me this treatment and for giving me a better quality of life.

## Lead author biography


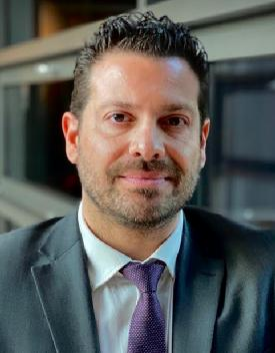
Dr Papadopoulos is a consultant cardiologist, head of Echocardiography department, specialized in advanced cardiac echo techniques (TOE, 3D, Stress Echo, Speckle tracking). He is experienced in guiding transcatheter interventions. He is a fellow of the ESC (FESC) and EACVI (FEACVI), and member of the EACVI Education, Scientific, TTE Accreditation and Training Grants committees. His PhD thesis was on MitraClip and deformation imaging and has several peer-reviewed publications. He was nominated with the “Education of Excellence and outstanding mid-career” award in 2025 during EACVI congress in Vienna.

## Supplementary Material

ytag630_Supplementary_Data

## Data Availability

The data underlying this article will be shared on reasonable request to the corresponding author.
